# Varied Presentation of Arrhythmogenic Right Ventricular Dysplasia/Cardiomyopathy (ARVD/C): A Case Series

**DOI:** 10.7759/cureus.33883

**Published:** 2023-01-17

**Authors:** Dhananjay Mishra, Om Shankar, Vikas Aggarwal

**Affiliations:** 1 Cardiology, Institute of Medical Sciences (IMS) Banaras Hindu University (BHU), Varanasi, IND

**Keywords:** ventricular tachycardia, cardiomyopathy, right bundle branch block, right ventricle, arvd

## Abstract

Arrhythmogenic right ventricular dysplasia (ARVD) is a genetically predisposed form of cardiomyopathy that mainly affects young individuals resulting in fatal ventricular arrhythmias leading to sudden cardiac death. ARVD has 50% of cases that involve both the right ventricle (RV) and left ventricle (LV), but only a small number of cases involve an isolated left ventricle. In this case series, five patients (four males and one female) with a diagnosis of ARVD presented to our center with varied clinical presentations across a wide range of age groups. The MRI of all five cases showed dilated right atrium (RA)/RV with right ventricular free wall dyskinesia. Two-dimensional (2D) MRI showed aneurysmal outpouching with diffuse free wall enhancement. Automated implantable cardioverter defibrillator (AICD) was implanted uneventfully in all five patients, and the patients were discharged with oral medications such as low-dose diuretics, beta-blockers, spironolactone, angiotensin-converting enzymes (ACE) inhibitors, amiodarone, and anxiolytics. Until now, the patients were doing well on follow-up visits. The therapeutic management of patients with arrhythmogenic right ventricular dysplasia/cardiomyopathy (ARVD/C) has evolved over the years and continues to be an important challenge. To further improve risk stratification and treatment of patients, more information is needed on natural history, long-term prognosis, and risk assessment. Special attention should be focused on the identification of patients who would benefit from implantable cardioverter-defibrillator (ICD) implantation in comparison to pharmacological and other nonpharmacological approaches.

## Introduction

Arrhythmogenic right ventricular dysplasia (ARVD) is a genetically predisposed form of cardiomyopathy that mainly affects young individuals resulting in fatal ventricular arrhythmias leading to sudden cardiac death, where malformed desmosomes fail to connect myocardial cells to each other leading to the dis-interruption of the propagation of electrical signal, which ultimately leads to the degeneration of myocardial cells, which is replaced by fibrofatty changes especially in the right ventricle (RV) that may progress further to involve the left ventricle (LV) [1,2].

ARVD has 50% of cases that involve both RV and LV, but only a small number of cases involve isolated left ventricular [3]. ARVD is frequently associated with ventricular tachycardia (VT) having left bundle branch block (LBBB) morphology and very rarely with VT having right bundle branch block (RBBB) morphology even in cases where left ventricles are involved [4]. ARVD is a progressive disease that may range from subclinical phase where the patients are mostly asymptomatic with subtle RV structural changes present but the risk of sudden cardiac death present. The next stage of the disease is overt electrical disorder where symptomatic RV arrhythmias prevail along with marked RV functional and structural abnormality present; following this stage, RV failure sets in with the progressive loss of RV myocardium and global RV dysfunction but with normal left ventricular function. The end stage of ARVD is biventricular failure where significant left ventricular dysfunction sets in leading to biventricular pump failure and to congestive heart failure [5]. The main cause of the progression of ARVD is sympathetic overstimulation.

The applied Task Force criteria give reasonable sensitivity for the diagnosis of ARVD, but there is not a single test developed to date that accurately diagnoses ARVD. Cardiac MRI holds diagnostic promises to diagnose this genetic cardiomyopathy, i.e., ARVD [6]. ARVD should be suspected in all young adults presenting with syncope, VT of LBBB morphology, and sudden cardiac death [7]. It causes sudden death in 30% of young adults and in 5% of those whose age is less than 65 years. In this case series, five patient with the diagnosis of ARVD presented to our center with varied clinical presentations across a wide range of age groups.

## Case presentation

Case 1

**Figure 1:**
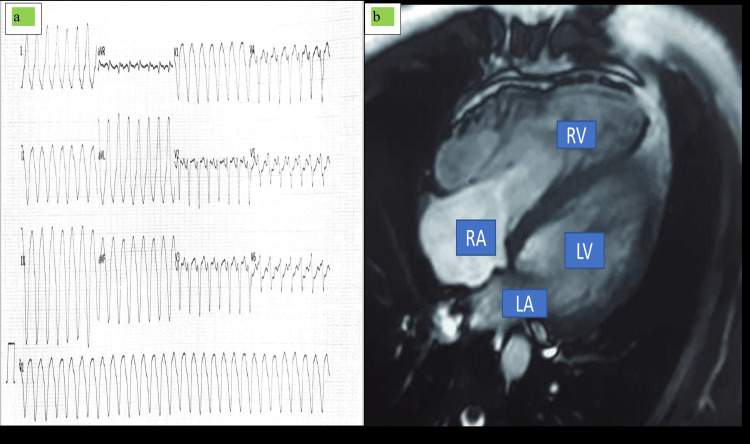
ECG and MRI findings of case 1. (a) ECG showed sustained ventricular tachycardia and left bundle branch morphology with the superior axis. (b) MRI showed dilated RA/RV free wall dyskinesia with diffuse free wall enhancement on gadolinium contrasts. RA, right atrium; RV, right ventricle; LA, left atrium; LV, left ventricle

A 21-year-old male presented to our center with a chief complaint of his heart racing inside his chest and a complaint of syncope. The patient was also complaining of multiple episodes of vomiting with cold profuse sweating with extreme anxiety and intense craving for cold water. On further inquiry, the patient denied any unexplained sudden death in his family. He also denied any history of substance abuse or any other form of drug abuse and any stress-related lifestyle changes. On examination, blood pressure was unrecordable, and pulse rate was very feeble. ECG tracing was taken out, which shows sustained ventricular tachycardia and left bundle branch morphology with the superior axis (Figure 1a). Multiple direct current (DC) cardioversion shocks of 200-360 J strength were given approximately 10-15 times and were managed accordingly for VT storm and to avoid further VT storm, amiodarone injection was given bolus in a dose of 150 mg intravenous (IV) slow over 10 minutes followed by 1 mg/minute for six hours and then 0.5 mg/minute for the next 18 hours; long-acting beta-blocker along with anxiolytics was added.

Repeat ECG after stabilization shows T wave inversion in lead V1-V4 with frequent ventricular premature contraction (VPC) and incomplete RBBB morphology with a leftward axis. Two-dimensional (2D) echocardiography (ECHO) was done, which shows dilated right atrium (RA)/RV with right ventricle outflow tract (RVOT) dimension of 38 mm and RV dysfunction (tricuspid annular plane systolic excursion {TAPSE}: 13 mm) with low-pressure severe tricuspid regurgitation and preserved left ventricular functions. Cardiac enzymes especially troponin I (3.52 ng/ml) were raised, which could be due to multiple DC cardioversions, or it may be due to acute coronary syndrome, so we planned for coronary angiography, which reveals a normal coronary artery. Based on the above clinical findings, it made us think of right-sided cardiomyopathy for we planned to go for cardiac MRI, which shows a nondilated left ventricle with an ejection fraction of 55% and dilated RA/RV free wall dyskinesia with diffuse free wall enhancement on gadolinium contrasts (Figure 1b). A definitive diagnosis of ARVD was made as per revised Task Force criteria (2020) [6]. The patient was counselled regarding prognosis and its fatal outcome of ventricular tachycardia/sudden cardiac death for which implantable cardioverter-defibrillator device (ICD-D) implantation was advised, which was implanted uneventfully, and the patient was discharged on oral medications such as low-dose diuretics, beta-blockers, spironolactone, angiotensin-converting enzyme (ACE) inhibitors, amiodarone, and anxiolytics, which was withdrawn over a two-week time with a continuation of other oral medications. The patient was doing well on follow-up (at four weeks) with adequate sleep and occasional few beats, which were agonizing in nature, but it got terminated by itself.

Case 2

**Figure 2:**
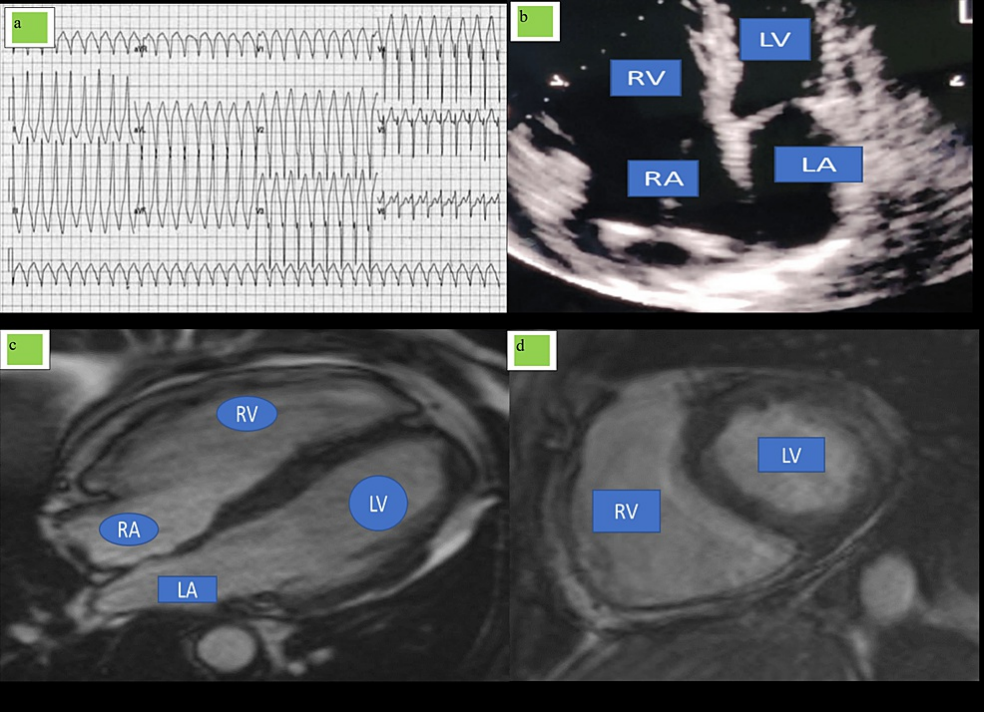
ECG, 2D ECHO, and MRI findings of case 2. (a) ECG showed frequent ventricular ectopy with incomplete RBBB morphology. (b) 2D ECHO showed the dilation of RA/RV with decreased right ventricular function. (c) MRI showed dilated RA/RV with right ventricular free wall dyskinesia. (d) MRI showed aneurysmal outpouching with diffuse free wall enhancement. 2D ECHO, two-dimensional echocardiography; RBBB, right bundle branch block; RA, right atrium; RV, right ventricle; LA, left atrium; LV, left ventricle

A 44-year-old male patient reported to our center with a complaint of recurrent syncope that was associated with recurrent fall with evidence of external injury on his body parts. The patient gave a history of palpitation, anxiety, loss of sleep, and undue stress on everyday activity, for which he was assessed by a local doctor and was diagnosed as having generalized anxiety disorder and was prescribed fluoxetine, propranolol, alprazolam, and olanzapine, and the patient improved partially with reference to anxiety and loss of sleep but started complaining of recurrent syncope. Patient was not having any history of hypertension, diabetes, and dyslipidemia; thyroid status was normal, and there was no family history suggestive of any heart disease or any history of sudden cardiac death in his family or first-degree relative. There was no habit of any form of tobacco abuse or alcohol or any high-risk behavior. He asserted to be occasional eater of nonvegetarian food items. The patient’s ECG was taken that showed frequent ventricular ectopy with incomplete RBBB morphology (Figure 2a). Patient was put on a 24-hour Holter monitoring, which shows 2:1 atrioventricular (AV) block with significant sinus pause of 2.2 seconds along with short run of ventricular tachycardia of LBBB morphology with superior axis.

A diagnosis of sick sinus syndrome seems to be likely, but during routine 2D ECHO examination, there was a dilation of RA/RV with decreased right ventricular function as TAPSE was 11 mm with associated right ventricle outflow tract obstruction (RVOTO) diameter of 40 mm and moderate low-pressure tricuspid regurgitation (right ventricular systolic pressure {RVSP}: 44 mm Hg), and the apical portion of right ventricular appears to be thinned out with akinesia in apical and free wall of right ventricles (Figure 2b). Left ventricular function was assessed to be normal without any reasonable wall motion abnormality and any associated abnormality of mitral and aortic valve. Brain natriuretic peptide (BNP) was 125 pg/ml with negative cardiac enzymes. Coronary angiography was normal. With reference to above intake, we thought that we are dealing with right-sided cardiomyopathy. Our center does not do cardiac muscle biopsy, so cardiac MRI was planned, which reveals dilated RA/RV with right ventricular free wall dyskinesia and aneurysmal outpouching with diffuse free wall enhancement; these cardiac MRI findings are suggestive of ARVD; based on the above findings, we approximate toward the diagnosis of ARVD as per Task Force criteria (2020) (Figure 2c-2d). Major focus was on the management part of the patients that can improve the quality of life and provide symptom-free states of syncope, non-sustained VT, and drugs for heart failure. We decided to implant automatic implantable cardioverter defibrillator-pacemaker (AICD-P) with antiarrhythmic drugs, diuretics, mineralocorticoids receptor antagonists, and ACE/angiotensin receptor blocker (ARB) inhibitors, and the patient was advised not to participate in any strenuous physical activity, and until the writing of this paper, the patient was doing well with regular follow-up.

Case 3

**Figure 3:**
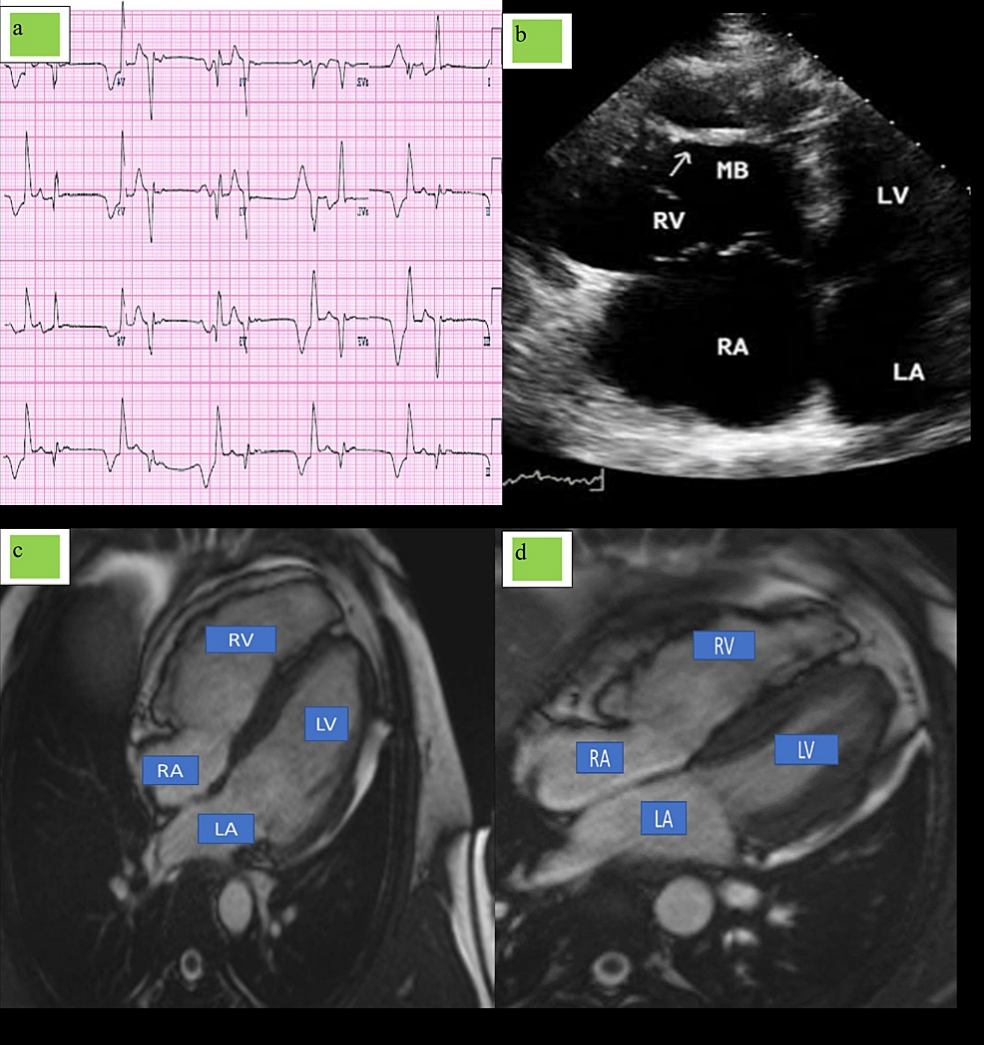
ECG, 2D ECHO, and MRI findings of case 3. (a) ECG showed frequent VPC with bigeminy, couplets, and T wave inversion in lead V1-V3 without RBBB. (b) 2D ECHO showed enlarged RA/RV with mild low-pressure tricuspid regurgitation. (c) MRI showed dilated right ventricle with RV dyskinesia/desynchrony. (d) MRI showed low-pressure tricuspid regurgitations. 2D ECHO, two-dimensional echocardiography; VPC, ventricular premature contraction; RBBB, right bundle branch block; RA, right atrium; RV, right ventricle; LA, left atrium; LV, left ventricle; MB, mitral bulge

A 40-year-old male who is an army by occupation came to our center with a complaint of chest pain on rest with palpitation mostly during rest or night. The patient was an occasional drinker and smokes occasionally and is involved in physical activity vigorously. There is no family history of any cardiac illness and sudden cardiac death. Frequent ECG tracing was taken, which showed frequent VPC with bigeminy, couplets, and T wave inversion in lead V1-V3 without RBBB (Figure 3a). A 24-hour Holter monitoring was planned, which does not reveal extra finding before and after the routine ECG. 2D ECHO was done, which showed enlarged RA/RV with mild low-pressure tricuspid regurgitation (RVSP: 25 mm Hg); right free wall looks relatively thinned out and hypokinetic, TAPSE was 14 mm, RVOTO dimension was 37 mm, and left ventricle ejection fraction (LVEF) was 58% by Simpsons methods (Figure 3b). With the above evidence of right-sided cardiomyopathy, we plan to go for cardiac MRI, which revealed dilated right ventricle with RV dyskinesia/desynchrony; right ventricle end diastolic volume index was 125 ml/m^2^, and right ventricle ejection fraction was 30% with cine evidence of low-pressure tricuspid regurgitations (Figure 3c-3d). AICD implantation was advised, which was done uneventfully, and the patient was discharged with amiodarone, metoprolol, diuretics, eplerenone, and ramipril. The patient was advised to stop any heavy physical activity with permits to do only daily pursuits, and the patient was advised to stop any heavy physical activity with permits to do only daily pursuits, and until the writing of this paper, patient was doing well and coming for monthly follow-up with occasional chest pain, which was of less intensity.

Case 4

**Figure 4:**
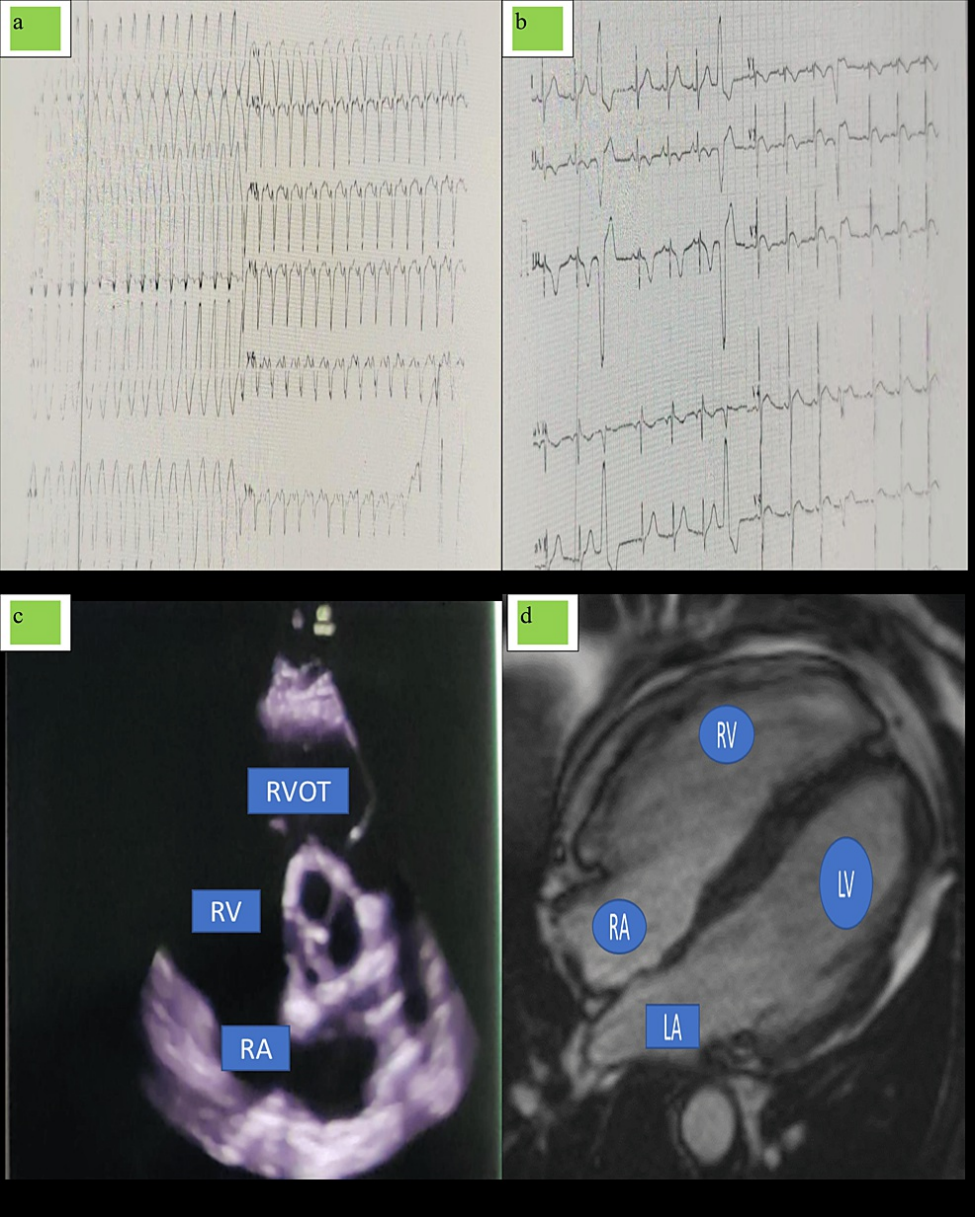
ECG, 2D ECHO, and MRI findings of case 4. (a) ECG showed ventricular tachycardia with LBBB and superior axis. (b) ECG showed the presence of T wave inversions in lead V1-V3 in the absence of complete RBBB and frequent premature ventricular rhythms. (c) 2D ECHO dilated RA and RV with normal right ventricular functions and moderate tricuspid regurgitation. (d) MRI showed outpouching of apical right ventricles with thinned right ventricle free wall and increased intensity of right ventricular free wall globally. 2D ECHO, two-dimensional echocardiography; LBBB, left bundle branch block; RBBB, right bundle branch block; RVOT, right ventricle outflow tract; RA, right atrium; RV, right ventricle; LA, left atrium; LV, left ventricle

A 26-year-old male presented with a complaint of palpitation, with extreme anxiety and profuse sweating and already diagnosed case of left-sided hemiparesis one month before for unexplained cause of ischemic stroke. On examination, pulse was very feeble, blood pressure was unrecordable, and the chest was clear with normal S1 and S2 present with no added abnormal heart sounds. ECG was taken, which shows ventricular tachycardia with LBBB and superior axis (Figure 4a). Electrical cardioversion was attempted with 200 J, 300 J, and 360 J with 8-10 attempts. Thinking it as a case of ventricular tachycardia storm, amiodarone injection was started, and intravenous 2 g of magnesium was given. Ventricular tachycardia was reverted back to normal sinus rhythms. The retrospective study of his previous ECG shows the presence of T wave inversions in lead V1-V3 in the absence of complete RBBB and frequent premature ventricular rhythms (Figure 4b). 2D ECHO was done, which shows dilated RA and RV with normal right ventricular functions (TAPSE of 22 mm with RVOTO diameter of 39 mm) and was also associated with moderate tricuspid regurgitation (RVSP: 54 mm Hg) (Figure 4c).

In light of the past history of sudden cardiac death of his elder brother, which was of unexplained etiology, we thought of hypertrophic obstructive cardiomyopathy as our first cause, but there was no asymmetrical hypertrophy of left ventricles or septum or any left ventricular outflow tract (LVOT) gradient. Coronary angiography was done to rule out the cause of ischemic ventricular tachycardia. Since the right chambers are enlarged, the etiology was most likely right-sided cardiomyopathy for which cardiac MRI was done. Cardiac MRI shows outpouching of apical right ventricles with thinned right ventricle free wall and was also associated with increased intensity of right ventricular free wall globally (Figure 4d). Right ventricular end diastolic volume index was 121 ml/m^2^, and right ventricular ejection fraction was 36%. Based on the above evidence, the patient was diagnosed to have ARVD. This patient has a history of revived ventricular tachycardia, occasional syncope, and first-degree relative dying of sudden cardiac death. The patient also has ischemic stroke leading to left-sided hemiparesis one month prior. To find out the cause of unexplained ischemic stroke, we did transesophageal echocardiogram (TEE) for patent foramen ovale and the examination of left atrial appendage, which was normal. Magnetic resonance (MR) angiography shows no abnormality of vasculature of the head and neck. The etiology of ischemic stroke is unknown in our case, but it may have any link with ARVD, which is questionable. Nevertheless, the patient with the diagnosis of ARVD was implanted with AICD uneventfully and was discharged with amiodarone, metoprolol, ramipril, and low-dose diuretics. The patient became apparently asymptomatic but was advised with three-month follow-up with timely compliance with the medicine.

Case 5

**Figure 5:**
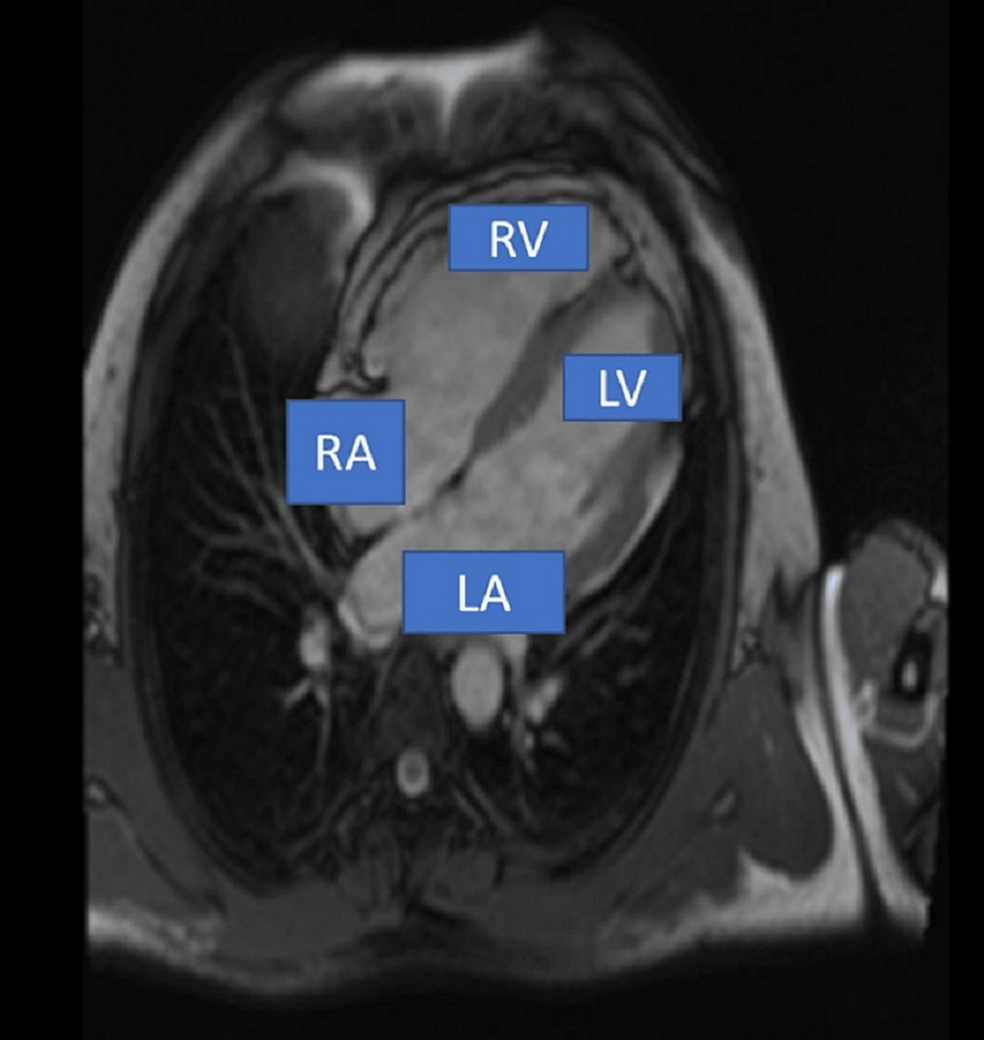
MRI showed grossly deranged right ventricular function with outpouching and thinned out apical segment with late gadolinium enhancement of right ventricular free wall. RA, right atrium; RV, right ventricle; LA, left atrium; LV, left ventricle

A 55-year-old female was complaining of occasional palpitation and syncope even at rest. She was a known diabetic for the last five years with well-controlled diabetic status on oral hypoglycemic drugs. She does not have a history of hypertension, hypothyroidism, bronchial asthma, chronic obstructive pulmonary disease (COPD), anxiety neurosis, etc. She had deranged lipid profile for which she was taking statins, i.e., increased low-density lipoprotein (LDL) of >130 mg/dl. ECG tracing shows few beats that do not have P-wave following every QRS complex with T wave inversion from V1-V4. Thinking that it may be a case of tachy-brady syndrome, a 24-hour Holter monitoring was done, which shows few episodes of atrial fibrillation that last for 10 seconds without any significant pause. 2D ECHO was done, which shows dilated RA/RV with low-pressure severe tricuspid regurgitation with right ventricular dysfunction (TAPSE: 12 mm) and thinning of right ventricles and akinetic right apical region that also involves right ventricular free wall. RVOTO diameter was 41 mm. Left ventricular function was 58% by Simpsons methods with trace mitral regurgitation, thick sclerotic aortic valve, and grade 1 diastolic dysfunction. This might be a case of RV cardiomyopathy for which cardiac MRI was done, which grossly deranged right ventricular function (RV ejection fraction was 34%) with outpouching and thinned out apical segment with late gadolinium enhancement of right ventricular free wall (Figure 5). Left ventricular ejection fraction was 50% with no regional wall motion abnormality. Based on the above findings, we made a diagnosis of ARVD with episodic atrial fibrillation for which AICD implantation was done; medical management includes amiodarone, metoprolol, diuretics, mineralocorticoids receptor antagonists, and warfarin therapy for six weeks. International normalized ratio (INR) was maintained around 2-3 for six weeks following discharge to avoid any thromboembolic complication due to atrial fibrillation. After six weeks, AICD interrogation was done to see the presence of atrial fibrillation; luckily, the intracardiac device interrogation fails to show atrial fibrillation from the day of implantation of AICD. Anticoagulant therapy was stopped with the continuation of other drugs, and the patient was advised for regular follow-up and proper drug compliance. She is doing well in the last one year with regular follow-up.

## Discussion

From the last 30 years, extensive study has been ongoing, which is defining the genetic etiology of ARVD in depth along with details of its pathogenesis and a broad spectrum of its clinical presentation. Different areas across the globe are reporting rare cases of ARVD with their varied presentation outside its classical presentation [8-12]. Recommendations for the standard diagnosis of patients with arrhythmogenic right ventricular dysplasia/cardiomyopathy (ARVD/C) based on ECG, and other factors (genetic, morphological, clinical, arrhythmic, and histopathologic) were published by the International Task Force (ITF) in 1994 and 2010 [13,14].

As the prevalence and incidence of ARVD are very low, there is no randomized study and adequate guideline to address the management protocol of ARVD. Treatment is based particularly on nonrandomized data, observational study, and expert opinion where more emphasis is given on individualized approach to treating rare diseases such as ARVD. The growing evidence of electrophysiology, arrhythmic outcome, growing therapeutic interventions such as AICD, antiarrhythmic drugs, catheter ablation, lifestyle changes, and risk factors make it particularly important to timely assess the case and place into perspective the issues relevant to the clinical management of this rare genetic disorder [13,14].

The estimated overall mortality rate varies among different studies, which ranges from 0.08% per year during a mean follow-up of 8.5 years in the case series by Nava et al. [15] to 3.6% per year during a mean follow-up of 4.6 years in the series by Lemola et al. [16].

The mechanism of sudden cardiac death in ARVD/C is cardiac arrest due to sustained ventricular tachycardia (VT) or ventricular fibrillation (VF), which may be the first presentation of the ARVD or sequelae of structural changes due to fibrofatty replacement of normal myocardium and overdrive sympathetic outflows [17-19].

Data from autopsy series and observational clinical studies on ARVD/C have provided a number of clinical predictors of adverse events and death such as cardiac arrest due to VT/VF, unstable sustained VT, and syncope due to arrhythmias or unrelated to arrhythmias; syncope unrelated to arrhythmias can be explained due to reflex-mediated changes in vascular tone or heart rate; reflexes such as micturition, defecation, coughing, and sneezing may precipitate nonarrhythmogenic syncope, which carries very poor outcome [20].

The chance of having a life-threatening arrhythmic episode is higher in patients who have sustained VT or VF [21,22]. In certain studies, but not all, unexplained syncope has been linked to an elevated arrhythmic risk [23].

Non-sustained VT on a 24-hour Holter monitoring and structural abnormalities such RV/LV insufficiency or dilation are additional independent risk factors for adverse outcomes. Also, among males when compared to females, there is unexplained and independent higher risk for the poor outcomes [24,25]. Other risk factors for poor outcome include heterozygosity (digenic or compound) in desmosomal gene mutation, early age at diagnosis, ventricular stimulation on programmed induction, the quantity of electroanatomic scar (related fractionated ECG), the degree of inversion of T wave inversion (especially in inferior and precordial leads), and QRS complex with low amplitude (including fragmentation) [25-29].

A useful diagnostic tool for differentiating between ARVD/C and other idiopathic RVOT tachycardias is the electrophysiological study (EPS). It also tests the susceptibility of induced ventricular tachycardia/ventricular fibrillation in electrophysiological (EP) laboratories, which may be fatal in daily life if left untreated. EPS also categorized the high-risk patient, which should be treated with AICD to avoid sudden cardiac arrest due to VT/VF, but in a multicentric study (with larger sample size) of patients with ARVD/C (ICD implantation), it was revealed that EPS is of minimal usefulness in the identification of patients at risk of arrhythmia-induced cardiac arrest as its predictive accuracy was low [20]. It was also observed in the above study that there was no significant prognostic difference between the patients being implanted with AICD for VT/VF based on their electrophysiological study and their controlled counterparts of ARVD not receiving AICD.

According to a research by Corrado et al., the positive predictive value (PPV) and negative predictive value (NPV) of inducibility for VT/VF were 35% and 70%, respectively, in 106 patients with ARVD/C who received an ICD for primary prevention [20]. A statistically different arrhythmic outcome over the follow-up period was not predicted in that study for the ventricular tachycardia/ventricular fibrillation induced in the EP catheterization laboratory. Inducible VT or VF at preimplant EPS did not indicate the need for suitable therapies for VT or VF during the course of a mean follow-up of 3.3 years, according to a study done on 98 patients with arrhythmogenic right ventricular cardiomyopathy (ARVC) in whom an ICD was implanted [22]. In the study by Bhonsale et al., a good number of patients were implanted with AICD despite the non-inducibility of VT/VF in EP laboratory but had episodes of VT/VF in the past. In this study, it was reported to have an indication of AICD implantation in asymptomatic individuals with a combination of more than two factors of the following: inducibility of VT/VF, proband status, non-sustained VT, and premature ventricular contractions (PVCs) of >1000/day [24].

Recent studies showed that the localization and quantification of RV scar area, as well as isolated island of surviving myocardium reflected as late potential, carry significant arrhythmogenic risk potential, which can be fatal. Endocardial voltage mapping (EVM) is a new addition to the EP study, which is an invasive procedure having additional arrhythmogenic risk potential for VT/VF [27].

Treatment of ARVD

Lifestyle changes include avoiding strenuous physical activity and competitive/endurance sports but doing recreational low-intensity sports. Competitive sports had been shown to increase the risk of sudden cardiac death by five times in young adult with the diagnosis of ARVD [30].

Antiarrhythmics such as class III agents are preferred and amiodarone in combination with beta-blockers. Beta-blockers must be added in all cases with the diagnosis of ARVD to counteract overstimulated sympathetic drive in all stages of disease progression ranging from asymptomatic individuals to biventricular failure, although its role in preventing sudden cardiac death is questionable [31,32].

Corrado et al. found that the majority of lifesaving AICD implantation was done in those high-risk patients who are already on antiarrhythmic drug therapy (AAD), which is indicated as an add-on therapy in patient with AICD or in patient with frequent VPC and in patient with non-sustained VT. These agents are also used in those patients without AICD being planned for catheter ablation as a backup procedure [30].

In genetically vulnerable mouse hearts, ventricular preload-reducing treatment delays the development of ARVD/C; according to experimental results presented by Fabritz et al., plakoglobin-deficient mice treated with nitrate and furosemide therapy did not develop enlarged RV due to training and had normalized inducibility for VT, resulting in phenotypically identical trained mouse (wild type). Heart failure medications (diuretics, ACE/ARB inhibitors, mineralocorticoid receptor antagonists {MRA}, and beta-blockers) are indicated in the patients presenting with right/left ventricular failure with the primary diagnosis of ARVD as they improve the symptoms of heart failure and retard the progression of ARVD [33,34].

In a retrospective study done by Wlodarska et al., they found that during a follow-up period (mean±SD) of 99±64 months, the incidence rate (annual) of complications related to thromboembolism in 126 patients with ARVD/C (having RV dilatation of severe nature) was 0.5%. Long-term oral anticoagulation is generally indicated in those patients who had documented evidence of intracavitary thrombus or any episode of thromboembolisms in the recent past. Prophylactic anticoagulation as a part of primary prevention of thromboembolism in those patients who had ventricular dilation or dysfunctions (regional/global) is not indicated [35].

Catheter ablation is a therapeutic option for ARVD patient with recurrent VT. The fibrofatty replacement of normal myocardium of RV/LV along with surviving island of myocardium creates an arrhythmogenic focus for VT/VF. VT is a result of a re-entry around the scar related to macro re-entry circuits, which can be mapped or located by electrophysiological study or substrate-based mapping [36,37].

The practicality of DC for the ablation of VT among the patients with ARVD/C was originally demonstrated by Fontaine et al. Later, a number of studies have discussed the immediate and long-term effects of VT ablation employing an endocardial catheter (using radiofrequency current) [36,37]. In general, 60%-80% of patients experienced immediate success; however, 50%-70% of patients experienced recurrence during long-term follow-up (three years to five years) [38-40]. Previously, endocardial catheter ablation showed remarkable degree of success, but it failed in few cases, but it was Garcia et al. who first observed the success of epicardial catheter in the ablation of VT in the patients with ARVD/C, who had undergone this approach after prior failure of VT ablation using endocardial catheter techniques [41].

Catheter ablation for the treatment of VT is indicated in patient with ARVD with incessant VT or in frequent VT episode while the patients are already on AICD. Epicardial catheter ablation is indicated only in failed attempts of endocardial ablation. It is also indicated in incessant VT or VT storm with failed drug therapy. AICD is the single best most efficacious mode of treatment for the management of ARVD. It is used as both primary and secondary prevention as ARVD is a progressive and degenerative disease and has the natural history that may present as VT/VF as initial symptoms leading to cardiac arrest and RV/LV dysfunction and ultimately leading to biventricular heart failure [23,41].

AICD implantation is a lifelong preventive measure and lifesaving intervention. AICD is indicated in high-risk group such as aborted sudden cardiac death, episodes of VT/VF, and severe ventricular dysfunctions. AICD implantation in these groups is most beneficial as life-threatening arrhythmic events are more than 10% per annum [23].

Among the patients with ARVD/C (LVEF of ≤35% and ECG showing wide QRS complex with LBBB pattern), cardiac resynchronization therapy (CRT) seems to be appropriate [42]. Regarding the clinical and hemodynamic consequences of RV pacing in the patients with ARVD/C (RV dysfunction and ECG showing wide QRS complex with RBBB pattern), it is still lacking [43]. Heart transplants are indicated as a treatment option for ARVD patients who have refractory heart failure or treatment-resistant tachyarrhythmias. A total of 18 patients with ARVD/C with 61% males and of mean age of 40±14 years received heart transplantation, according to Tedford et al.’s report [44].

Recommendations

While diagnosing/evaluating the patients with suspected ARVD/C (class IIa), EPS should be taken into consideration. For the classification purpose of arrhythmic risk among patients with asymptomatic ARVD/C (class IIb), programmed ventricular stimulation (PVS) may be taken into consideration (class IIb). The patients with ARVD/C (class IIb) may be evaluated both diagnostically and prognostically using endocardial voltage mapping (EVM) (class IIb) [45].

The patients with ARVD/C (class I) who have had at least one episode of VT or VF (sustained and hemodynamically unstable) are indicated to have an ICD implanted. Regardless of arrhythmias, ICD implantation is advised in patients with ARVD/C (severe systolic dysfunction of either or both of RV/LV) (class I). The patients with ARVD/C (class IIa) who have had at least one episode of VT or VF (sustained and hemodynamically stable) can be considered to have an ICD implanted. ARVD/C patients (class IIa) with “significant” risk factors (non-sustained VT/unexplained syncope/mild ventricular dysfunction) should be having an ICD implanted. After carefully weighing the risks (long term) and advantages of ICD implantation, the patients with ARVD/C (class IIb) with “minimal” risk factors may consider having an ICD implanted (class IIb) [45].

## Conclusions

Managing ARVD/C patients effectively has changed over time and remains a significant concern. More data on the natural history, risk assessment, and long-term prognosis are needed in order to strengthen the stratification of risk and patient therapy. The selection of patients who will benefit from the implantation of ICD when compared to pharmacological/nonpharmacological methods should receive special attention. To give the best possible evidence-based suggestions for the treatment of patients with ARVD/C, findings from randomized controlled trials (RCTs) of multicentric nature, from institutional registries, or from studies of prospective nature with a large enough sample size and a follow-up period of several years are desired. Existing treatment approaches are palliative rather than curative. The understanding of the molecular pathways underlying the etiology and pathogenesis of ARVD/C will serve as the basis for the design of a therapeutic intervention.
